# Evolutionary Analysis of HIV-1 Pol Proteins Reveals Representative Residues for Viral Subtype Differentiation

**DOI:** 10.3389/fmicb.2017.02151

**Published:** 2017-11-02

**Authors:** Shohei Nagata, Junnosuke Imai, Gakuto Makino, Masaru Tomita, Akio Kanai

**Affiliations:** ^1^Institute for Advanced Biosciences, Keio University, Tsuruoka, Japan; ^2^Faculty of Environment and Information Studies, Keio University, Fujisawa, Japan; ^3^Systems Biology Program, Graduate School of Media and Governance, Keio University, Fujisawa, Japan

**Keywords:** HIV-1, bioinformatics, pol protein, protein domain, network analysis, molecular evolution

## Abstract

RNA viruses have been used as model systems to understand the patterns and processes of molecular evolution because they have high mutation rates and are genetically diverse. *Human immunodeficiency virus 1* (HIV-1), the etiological agent of acquired immune deficiency syndrome, is highly genetically diverse, and is classified into several groups and subtypes. However, it has been difficult to use its diverse sequences to establish the overall phylogenetic relationships of different strains or the trends in sequence conservation with the construction of phylogenetic trees. Our aims were to systematically characterize HIV-1 subtype evolution and to identify the regions responsible for HIV-1 subtype differentiation at the amino acid level in the Pol protein, which is often used to classify the HIV-1 subtypes. In this study, we systematically characterized the mutation sites in 2,052 Pol proteins from HIV-1 group M (144 subtype A; 1,528 subtype B; 380 subtype C), using sequence similarity networks. We also used spectral clustering to group the sequences based on the network graph structures. A stepwise analysis of the cluster hierarchies allowed us to estimate a possible evolutionary pathway for the Pol proteins. The subtype A sequences also clustered according to when and where the viruses were isolated, whereas both the subtype B and C sequences remained as single clusters. Because the Pol protein has several functional domains, we identified the regions that are discriminative by comparing the structures of the domain-based networks. Our results suggest that sequence changes in the RNase H domain and the reverse transcriptase (RT) connection domain are responsible for the subtype classification. By analyzing the different amino acid compositions at each site in both domain sequences, we found that a few specific amino acid residues (i.e., M357 in the RT connection domain and Q480, Y483, and L491 in the RNase H domain) represent the differences among the subtypes. These residues were located on the surface of the RT structure and in the vicinity of the amino acid sites responsible for RT enzymatic activity or function.

## Introduction

*Human Immunodeficiency Virus 1* (HIV-1) is a retrovirus, a specific type of RNA virus that has been widely used as a model system for studying the molecular evolution of life because it is highly adaptive and highly genetically diverse. HIV-1 has a single-stranded RNA genome and synthesizes double-stranded DNA based on its RNA genome using reverse transcriptase (RT), which is retained within the viral particle after it enters the target cell. The HIV-1 genome contains nine genes: *gag*, encoding the structural proteins involved in viral particle formation; *env*, encoding the envelope protein; *pol*, encoding the enzymes for replication (protease, RT, RNase H, integrase); *tat* and *rev*, involved in the regulation of gene expression; and *vif*, *vpr, vpu*, and *nef*, which are accessory genes required for optimal viral replication *in vivo*.

Molecular phylogenies have shown that HIV-1 arose in humans by cross-species infection from chimpanzees at the beginning of the twentieth century (Sharp and Hahn, 2010), and the infection has spread worldwide since the latter half of the twentieth century. This lineage, which is the predominant lineage throughout the world, is called group M and is classified into nine subtypes based on their phylogenetic relationships: subtypes A, B, C, D, F, G, H, J, and K. This genetic diversity is mainly attributed to an error-prone RT (Preston et al., 1988) and the genetic recombination mechanism of retroviruses (Hu and Temin, 1990). Recombination occurs frequently between the same subtypes or between different subtypes, and plays an important role in the diversification of HIV-1 (Rambaut et al., 2004).

The rates of disease progression and transmission differ according to the HIV-1 subtype involved, and it is thought that these differences contribute to differences in the prevalence and expansion of the subtypes. Several studies have reported that subtype D infections have a faster disease progression rate than subtype A infections (Kaleebu et al., 2002; Vasan et al., 2006; Baeten et al., 2007; Kiwanuka et al., 2008; Ng et al., 2013); the transmissibility of subtype C is greater than that of subtype A or D (Renjifo et al., 2004); the replication capacity of subtype C is lower than that of the other group M subtypes (Abraha et al., 2009; Kiguoya et al., 2017); and RT activity during replication differs between subtypes B and C (Armstrong et al., 2009; Iordanskiy et al., 2010). Several studies have also detected sequence differences in the HIV-1 proteases and the active N-terminal regions of RT and integrase (Gordon et al., 2003; Kantor et al., 2005; Rhee et al., 2006; Myers and Pillay, 2008). The Pol protein, which contains these regions, is thought to be associated with the differences in the replication capacity and disease progression of the different subtypes (Ng et al., 2013). However, the functional regions or amino acid residues in each viral protein that correspond to subtype differentiation have not been clarified.

The HIV-1 subtypes have usually been classified according to phylogenetic trees based on nucleotide or protein sequences of the viral core genes (*gag, pol*, and *env*; Castro-Nallar et al., 2012) and the clade relationships established (Robertson et al., 2000). Phylogenetic trees reflect the bifurcating phylogenetic relationships of sequences, but the construction of exact trees is difficult when the sequences contain intrasubtype recombinants, which occur frequently in HIV-1 (Posada et al., 2002; Arenas and Posada, 2010). Therefore, we constructed a sequence similarity network (SSN), a weighted undirected graph based on sequence similarities, to visualize the sequence space and observe the positional relationships among the subtypes in various regions of the HIV-1 genome.

Our aims were to systematically characterize the evolution of the HIV-1 subtypes, and to clarify the sequence regions that are responsible for the differentiation of the viral subtypes. In this study, we analyzed the mutation sites in 2,052 Pol proteins from HIV-1 group M using SSNs. Because the Pol protein is often used for group or subtype classification, we determined the overall positional relationships among the subtypes based on the Pol sequences. We then compared the structures of the domain-based networks to identify the regions that characterize the subtypes. The amino acid sites corresponding to the different subtypes were specified and mapped to the three-dimensional structure of the protein. We discuss the possible implications of these results in light of the kinds of regions that have changed during the adaptation of the virus in its spread throughout the world.

## Materials and methods

### Data sources

The near-complete genome sequences and their attributions (sampling year, sampling region, and subtype/sub-subtype of the viral sequence) of HIV-1 group M subtypes A (which consists of sub-subtypes A1 and A2), B, and C were downloaded from the HIV Sequence Database at Los Alamos (http://www.hiv.lanl.gov, last accessed August 2014). We used these three subtypes because the genetic distances between their sequences are almost equivalent (Robertson et al., 2000) and the number of sequences registered in the database is large enough for our analysis. After the intersubtype recombinants and truncated sequences were excluded, 2,052 Pol protein sequences (144 subtype A; 1,528 subtype B; 330 subtype C) were obtained. The regional breakdown of the datasets is shown in Table 1.

**Table 1:** Geographic regional breakdown of HIV-1 datasets used in this study.

	**Number of sequences**						
**Subtype**	**Africa**	**Asia**	**Central and South America**	**Europe**	**North America**	**Oceania**	**Total**
A	73 (1)	38 (1)	0	31	0	2	144 (2)
B	2	222	149	148	985	22	1,528
C	338	28	6	6	2	0	380

*Subtype A can further be divided into sub-subtypes A1 and A2; the numbers in parentheses show the number of sub-subtype A2 sequences*.

### Network analysis based on sequence similarities

The sequence similarity scores were calculated to construct a weighted undirected graph (SSN). The similarity scores (Basic Local Alignment Search Tool [BLAST] bit scores; Altschul et al., 1990) for all the HIV-1 Pol protein sequences were calculated with an all-against-all BLASTP (BLAST 2.2.31+) analysis (Altschul et al., 1997; Camacho et al., 2009), with a cut-off *E*-value of ≤ 1e−5. Using the BLAST bit scores, the sequence similarities were normalized to 0.0–1.0, with the following equation (Dufour et al., 2010; Matsui et al., 2013):

$$\begin{array}{l} sim\left(x,y\right)=\frac{max\;\left(bit\;score\;\left(x,y\right),\;bit\;score\;\left(y,x\right)\right)}{max\;\left(bit\;score\;\left(x,x\right),\;bit\;score\;\left(y,y\right)\right)} \end{array}$$

where sim(*x*,*y*) represents the normalized sequence similarity between two sequences *x* and *y*. If the score was 1.0, the pair was deemed to be identical. A weighted undirected graph was constructed based on the scores of all the pairs of sequences, and the edges were weighted with the scores. We set a threshold sequence identity value and connected the nodes when the sequence identity exceeded the threshold. The threshold to be used was determined by comparing the networks constructed with an incremental series of threshold values. We constructed SSNs of both the full-length Pol protein sequences and the functional domain sequences within the Pol protein. The constructed networks were visualized with Cytoscape 3.4.0 (Shannon et al., 2003), with a force-directed layout.

### Clustering based on the network structure

Spectral clustering (Paccanaro et al., 2006), a clustering method that divides data into clusters based on the structure of a network graph, was performed with SCPS 0.9.5 (Nepusz et al., 2010) for the networks constructed from the full-length Pol protein sequence. With this clustering algorithm, we analyzed the factors (sampling year, sampling region, and subtype) that affected the mutations in the Pol proteins by gradually changing the number of divisions.

### Extraction of functional domain sequences in the Pol protein

Based on the HIV-1 group M subtype B reference strain HXB2 (GenBank accession: K03455), information on the functional domains was obtained from the Swiss-Prot database (http://www.uniprot.org/; The UniProt Consortium, 2017), a high-quality annotated protein sequence knowledgebase. The dataset sequences were then aligned to the HXB2 Pol sequence using MAFFT L-INS-i 7.245 (Katoh and Standley, 2013) to extract the functional domains. Note that the amino acid residues mentioned in this study are numbered according to the HXB2 sequence.

### Construction of phylogenetic trees from functional domain sequences of Pol protein

We randomly selected 10 sequences from each subtype (subtype A, B, or C) to represent each functional domain sequence. A multiple-sequence alignment of each domain was created with MAFFT L-INS-i 7.245 (Katoh and Standley, 2013), and maximum likelihood phylogenetic trees were constructed with RAxML 8.2.9 (Stamatakis, 2014; GAMMA model with 1,000 bootstrap replicates). The calculated trees were visualized with FigTree 1.4.2 (http://tree.bio.ed.ac.uk/software/).

### Calculation of cumulative relative entropy (CRE)

To identify the sites that characterize the differences between each subtype of HIV-1, we calculated CRE (Hannenhalli and Russell, 2000) for each amino acid site in the Pol protein. We calculated the amino acid compositions of the three subtypes (A, B, and C) at each site in a multiple alignment. The *hmmbuild* program of HMMER 3.1b2 (Eddy, 1998) was used to build profile P of the alignment. The weighting method of Henikoff and Henikoff (1994) was used for the residue counts. The relative entropy (Shannon, 1996; Durbin et al., 1998) of position *i* for subtype $\bar{s}$ with respect to the entropy of that position for subtype was calculated. If ${\text{RE}}_{i}^{s}$ is the relative entropy of ${\text{P}}_{i}^{s}$ with respect to P_*i*_:

$$\begin{array}{l} RE_{i}^{s}=\underset{for\;all\;x}{\sum }P_{i,x}^{s}\log\frac{P_{i,x}^{s}}{P_{i,x}^{\bar{s}}} \end{array}$$

Note that RE is greater than or equal to zero, and is exactly zero when the two distributions are identical (Durbin et al., 1998). To estimate the role of alignment position *i* in characterizing the HIV-1 subtypes, CRE_*i*_ was calculated as:

$$\begin{array}{l} CRE_{i}=\;\underset{for\;all\;subtypes\;s}{\sum }RE_{i}^{s} \end{array}$$

The CREs for all the positions were converted into Z-scores based on the distribution of the entropies within a sequence alignment. Let μ and σ be the means and standard deviations of the CREs of all positions, then the Z-score for position *i* is calculated as:

$$\begin{array}{l} Z_{i}\;=\;\frac{CRE_{i}-\;\mu }{\sigma } \end{array}$$

We expect a position with a high Z-score to be important in characterizing the subtypes. We calculated the CREs, and positions with Z-scores of CRE ≥ 3.0 were defined as “high-CRE” positions. All multiple sequence alignments used to calculate CRE were constructed with MAFFT L-INS-i 7.245 (Katoh and Standley, 2013). The generated alignments were visualized and sequence conservation was calculated with Jalview 2.9.0b2 (Waterhouse et al., 2009).

### Protein conformation analysis of RT

The functional domains characterizing the subtypes and the high-CRE residues were mapped onto the structure of HIV-1 RT. The protein structural data (PDB ID: 1REV; 3KJV for RT complexed with the DNA duplex) were obtained from the Protein Data Bank (http://www.rcsb.org/pdb; Bernstein et al., 1977), a database of experimentally determined protein structures, and visualized with UCSF Chimera 1.10.2 (Pettersen et al., 2004).

## Results

### Comparison of thousands of HIV-1 Pol sequences based on a network analysis

**Figure 1:**
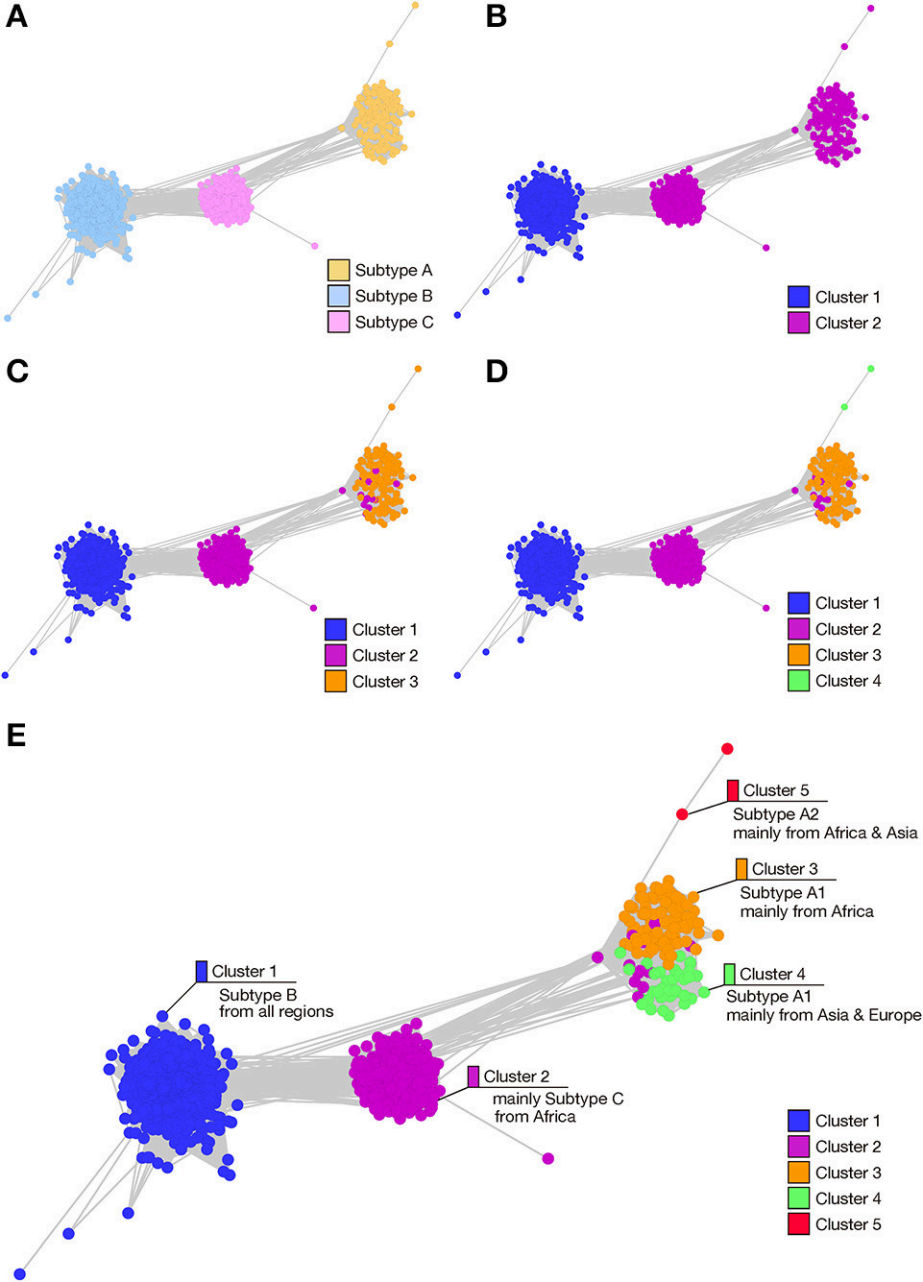
Stepwise analysis of the SSNs of HIV-1 Pol protein. In total, 2,052 amino acid sequences of the HIV-1 Pol protein were used to construct networks based on sequence similarities (SSNs). Nodes (colored dots) represent the Pol protein sequences and the edge lengths represent the sequence similarities. **(A)** Nodes are colored according to the HIV-1 subtype. **(B–E)** To investigate the possible process of Pol protein evolution, a total of 2,052 Pol sequences, used to construct the network structure shown in panel A, were clustered into 2, 3, 4, or 5 groups with the spectral clustering method by changing the number of divisions in a sequential order, and are colored according to cluster. A stepwise analysis of the hierarchy of the clusters allowed the possible evolutionary pathways of the Pol proteins to be estimated. Panel **(E)** shows the attributes (subtype, region of sampling) of the sequences in each cluster.

The classification of and relationships between each HIV-1 subtype were determined by constructing networks based on the amino acid sequence similarities of the Pol polyprotein (Supplementary Figure 1 and Figure 1). The SSN is a graphical representation of the similarities between sequences. Each sequence is indicated by a point (node) and the similarity between the sequences is represented by the length of the line (edge) connecting the points. The smaller the distance between the nodes, the greater the degree of similarity between the sequences. We used subtypes A, B, and C from HIV-1 group M in the present analysis. Subtypes A, B, and C clearly form distinct groups when their sequence similarities are analyzed (Robertson et al., 2000) and more of these sequences are registered in the database than those of other subtypes, so we assumed that enough sequences were available for our purpose.

When constructing an SSN, the network structure changes according to the threshold value of the sequence identity used when connecting the edges (Fujishima et al., 2008). Therefore, we first constructed a series of networks by gradually changing the threshold value and compared their structures (Supplementary Figure 1). In networks in which the edges were connected with sequence identities ≥80%, the three subtypes of HIV-1 were not well-separated and formed one large network (Supplementary Figure 1A). When the sequence identity threshold was ≥92%, each of the three subtypes was properly separated. Because the nodes were still connected under this threshold, the relative positional relationships among subtypes were determined (Supplementary Figure 1B). However, as the threshold value became much stricter, the connections between the subtypes were broken, and with a sequence identity threshold of ≥96%, more than 130 graphs were generated, so that it was impossible to determine the exact positional relationships among the subtypes. Therefore, we adopted a threshold value for sequence identity of ≥92% for the subsequent analysis.

In Figure 1A, the nodes are colored to represent the three subtypes shown in Supplementary Figure 1B, and provides an overall view of the sequence similarities between and/or within each subtype. To confirm the subtype groupings in the same SSN and to clarify the attributions obtained from the database (sampling year, sampling region, and viral sequence subtype), we used the spectral clustering method, which divides sequence data into clusters based on the structure of a network graph. This methodology enables the estimation of the process of subtype differentiation according to the order of cluster division when dividing clusters step by step. Therefore, we changed the clustering division numbers to two clusters (Figure 1B), three clusters (Figure 1C), four clusters (Figure 1D), and five clusters (Figure 1E). At the two-cluster stage (Figure 1B), subtype B and subtypes A and C formed specific groups. Subtypes A and C were then differentiated into different groups in the three-cluster stage (Figure 1C). These evolutionary steps are consistent with the order of subtype differentiation reported in a previous study (Castro-Nallar et al., 2012), and the elements of the clusters and each subtype in the three-cluster stage showed a high coincidence ratio (99.4%), indicating that this network analysis is a suitable technique for classifying these subtypes (Figures 1A,C). When the number of divisions was set to 4, the sequence group corresponding to subtype A was further divided into two clusters, which exactly matched sub-subtypes A1 and A2, respectively (Figure 1D). With five clusters, subtype A1 was further divided into an additional two clusters, consisting mainly of the sequences from Africa or from Asia and Europe (Figure 1E).

### Domain-based network analysis shows that RNase H domain and RT connection domain are important for subtype differentiation

**Figure 2:**
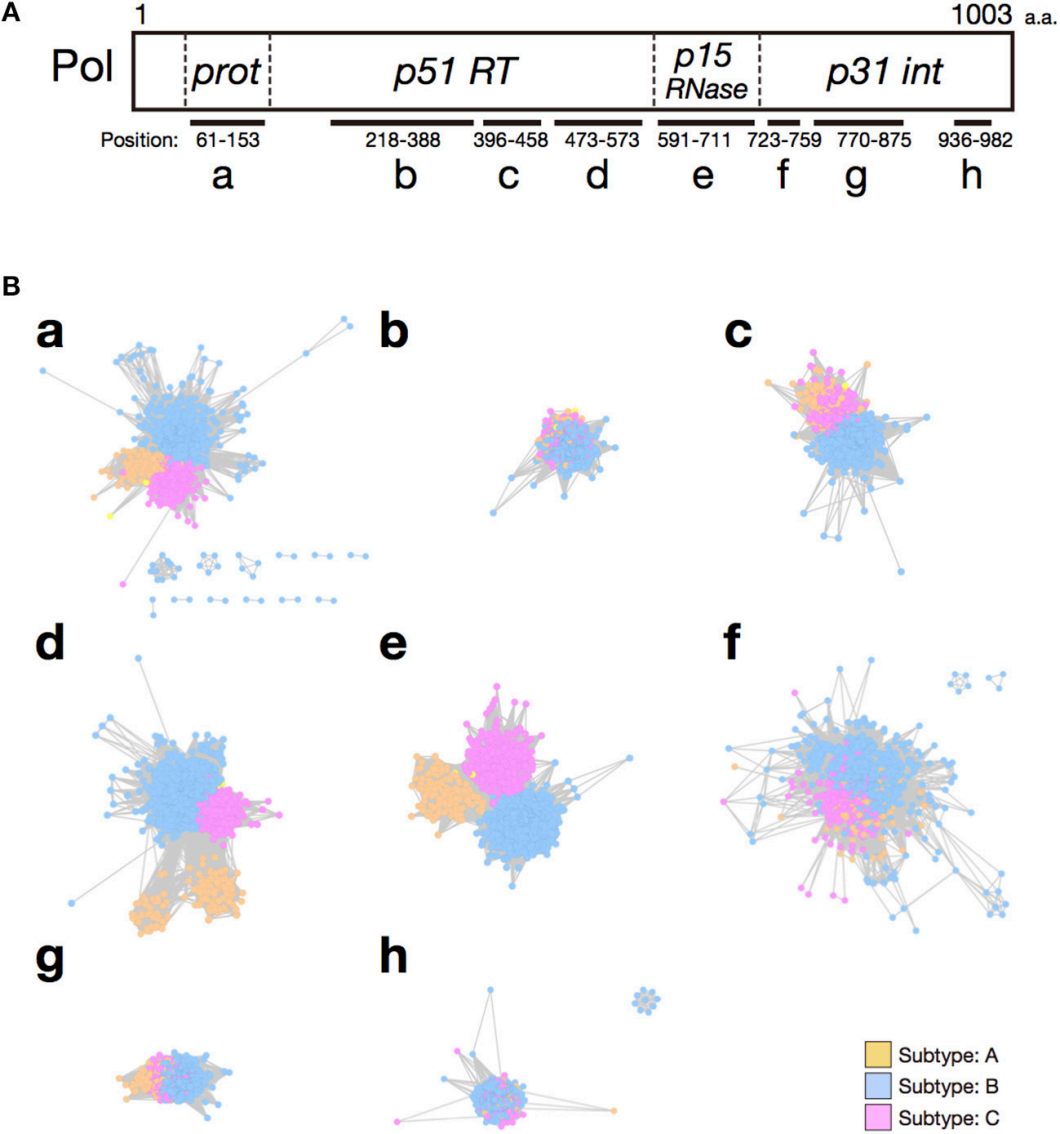
Comparison of the SSNs of functional domains of the HIV-1 Pol protein. **(A)** Schematic representation of the positions and lengths of the functional domains of the HIV-1 Pol protein: **a**, retroviral aspartyl protease; **b**, RT (RNA-dependent DNA polymerase); **c**, RT thumb domain; **d**, RT connection domain; **e**, RNase H; **f**, integrase zinc-binding domain; **g**, integrase core domain; and **h**, integrase DNA-binding domain. **(B)** SSNs of each functional domain region of the HIV-1 Pol protein (2,052 sequences) were created and colored according to subtype. Nodes represent the sequences of each functional domain and the edge lengths represent the sequence similarities. Symbols **(a–h)** correspond to those in panel **(A)**.

To analyze the regions in the HIV-1 Pol polyprotein that are responsible for HIV-1 subtype differentiation, we constructed SSNs based on each functional domain (Figure 2). Figure 2A shows the eight domains present in the HIV-1 Pol polyprotein (**a**, retroviral aspartyl protease, residues 61–153; **b**, RT (RNA-dependent DNA polymerase), residues 218–388; **c**, RT thumb domain, residues 396–458; **d**, RT connection domain, residues 473–573; **e**, RNase H, residues 591–711; **f**, integrase zinc-binding domain, residues 723–759; **g**, integrase core domain, residues 770–875; and **h**, integrase DNA binding domain, residues 936–982). When we compared the SSNs constructed for each domain, the nodes of the three subtypes were mixed in the networks of the RT (RNA-dependent DNA polymerase) region (Figure 2Bb) and the integrase DNA-binding domain (Figure 2Bh) because of the high sequence conservation in these regions. In the RT thumb domain (Figure 2Bc), the integrase zinc-binding domain (Figure 2Bf), and the integrase core domain (Figure 2Bg), the nodes for subtype A and C were mixed, although those for subtype B and subtypes A and C were separated. Therefore, we consider these regions unsuitable for distinguishing these subtypes. In contrast, the nodes of the three subtypes were well-separated in another three domains: the retroviral aspartyl protease region (Figure 2Ba), the RT connection domain (Figure 2Bd), and the RNase H domain (Figure 2Be). Among these, we consider the retroviral aspartyl protease region inappropriate for distinguishing the subtypes because the nodes are dispersed compared with those of the other two regions. Therefore, we conclude that the RT connection domain and the RNase H domain represent the differences among the HIV-1 subtypes. To observe effects of non-domain regions on subtype classification, we extracted five regions lying between the eight domains of the Pol protein (Supplementary Figures 2Ai–m) and constructed the corresponding SSNs (Supplementary Figure 2B). However, in these networks of non-domain regions, the boundaries of the three subtypes were indefinable. In particular, for the region shown in Supplementary Figure 2Ai, hundreds of graphs were generated, which did not provide clear indices for distinguishing the subtypes.

**Figure 3:**
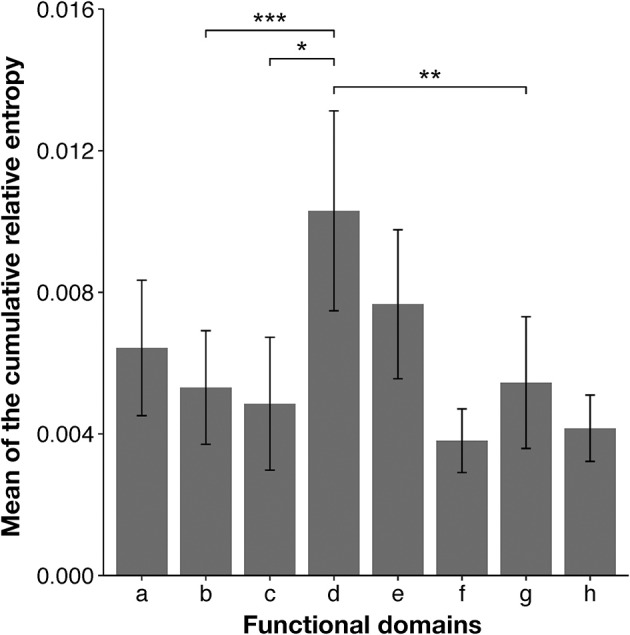
Mean cumulative relative entropy (CRE) values for each functional domain of HIV-1 Pol protein. The x-axis represents each functional domain region, corresponding to those in Figure 2A: a, retroviral aspartyl protease; b, RT (RNA-dependent DNA polymerase); c, RT thumb domain; d, RT connection domain; e, RNase H; f, integrase zinc-binding domain; g, integrase core domain; and h, integrase DNA-binding domain. The y-axis represents the mean CRE value per site in each domain region. Groups were compared with the Mann–Whitney *U-*test (^*^P < 0.05; ^**^P < 0.01; ^***^P < 0.001). Error bars represent standard errors of the means and asterisks indicate the significance of differences between the RT connection domain or RNase H domain and each region.

To confirm these results, we analyzed each selected domain phylogenetically, with the maximum likelihood method. The results supported our conclusions based on our domain-based SSNs (Supplementary Figure 3). In the phylogenetic trees for the RT connection domain (Supplementary Figure 3A) and the RNase H domain (Supplementary Figure 3B), branches of the three subtypes are clearly separated. However, in other domains, such as the RT (RNA-dependent DNA polymerase) domain (Supplementary Figure 3C) and integrase core domain (Supplementary Figure 3D), there are ambiguous boundaries among the nodes corresponding to each subtype. In particular, the nodes of the three subtypes are mixed in the clades for the integrase zinc-binding domain (Supplementary Figure 3E). Furthermore, to quantitatively identify the domains of the sequences that characterize the differences among subtypes, CRE was calculated for each site after the sequences were aligned. This index takes a large value when the difference between an amino acid distribution in one subtype and that in the other subtypes is large. The mean CRE value was calculated for each domain of the Pol protein. The regions with the highest average CRE values were the RT connection domain and the RNase H domain (Figure 3). This supports the results based on the network structures shown in Figure 2B. The mean CRE value for the RT connection domain was statistically significantly larger than those for the other regions.

### Mapping the amino acid residues that are crucial for subtype specification

**Figure 4:**
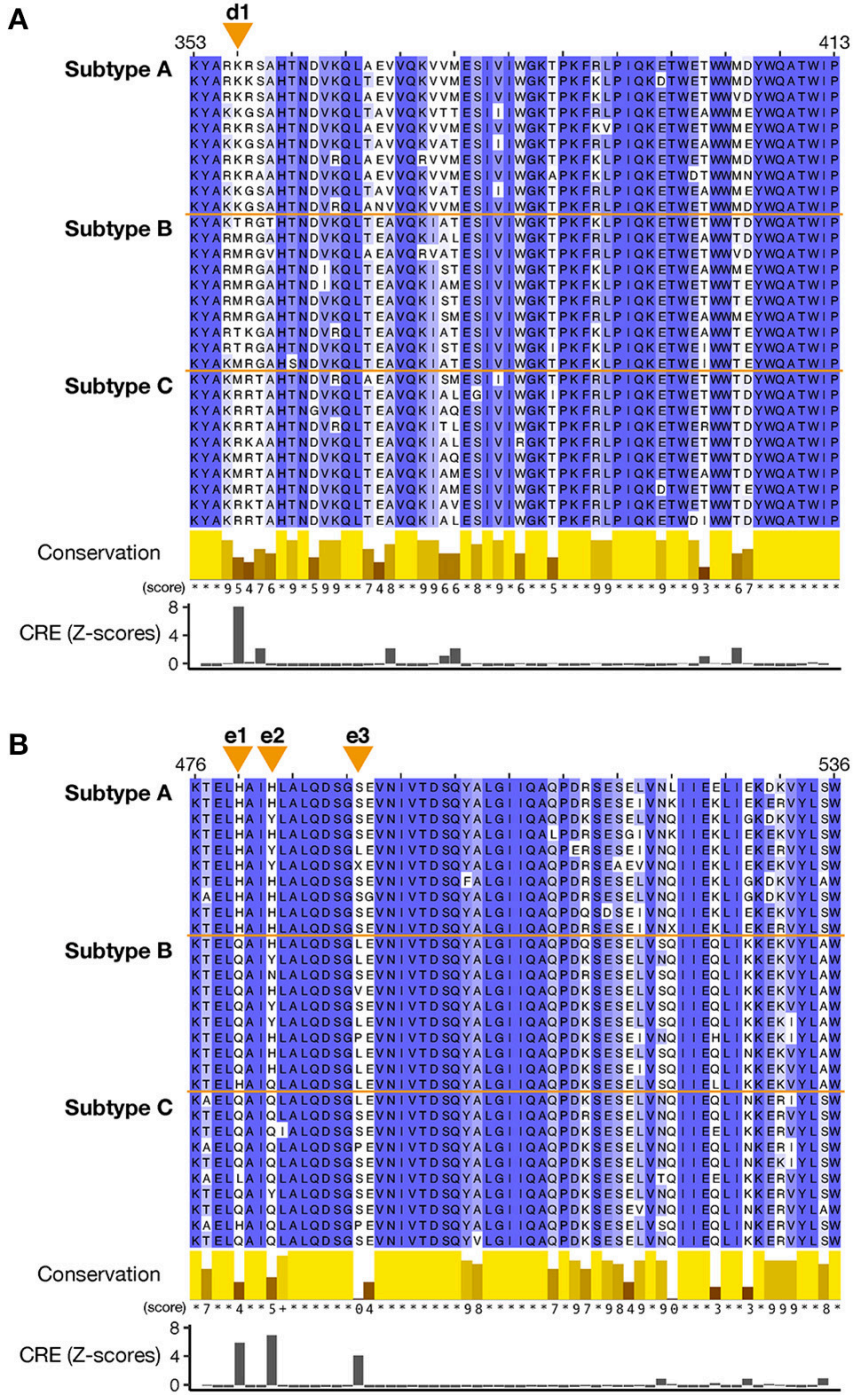
Multiple amino acid sequence alignments of the RT connection domain and RNase H domain of the Pol protein. A total of 30 sequences, 10 sequences randomly selected from each subtypes (subtypes A, B, and C), were aligned with the MAFFT L-INS-i algorithm. **(A)** Sequence alignment around the sites in the RT connection domain that characterize the subtypes. **(B)** Sequence alignment around the sites in the RNase H domain that characterize the subtypes. For each amino acid position, the conservation scores according to Livingstone and Barton (1993) are shown as 12 ranks (from 0 to 11). Identical amino acids (rank 11) are indicated by asterisks, and partly conserved amino acids (rank 10) are indicated by a plus symbol. Orange triangles at the top of each alignment indicate the sites with CRE Z-scores ≥ 3.0 (designated d1 and e1-3). CRE was calculated based on all the sequences in the dataset. Sequence conservation scores and Z-scores of CRE for each site are shown together at the bottom of each alignment.

To clarify the changes in the amino acid residues that distinguish the three subtypes, the CRE value was calculated for each site in both the RT connection domain and RNase H domain. The sites with CRE-derived Z-scores ≥ 3.0 (see section Materials and Methods) were then identified on an amino acid sequence alignments (Figure 4). High-CRE sites occurred at position 357 in the RT connection domain (Figure 4A) and at positions 480, 483, and 491 in the RNase H domain (Figure 4B). At position 357 (d1 site on Figure 4A) in the RT connection domain, lysine (K) occurs in 100% of subtype A sequences, methionine (M) in the majority (70%) of subtype B sequences, and methionine (M) and arginine (R) in about 50% each of the subtype C sequences, so the amino acid distribution at this site differs in the three subtypes. Meanwhile, in the RNase H domain, the amino acid compositions of subtypes B and C are similar at position 480 (e1), those of subtypes A and B are similar at position 483 (e2), and those of subtypes A and C are similar at position 491 (e3). In the RT connection domain, the subtypes can be distinguished at one site, whereas in the RNase H domain, the subtypes can be distinguished at three sites. These three sites are situated in close proximity in the RNase H domain. In terms of the sequence conservation in both domains, a region with low conservation is distributed throughout the domains, but these regions are not necessarily high-CRE regions. Therefore, we conclude that low sequence conservation does not generate the differences between the subtypes.

**Figure 5:**
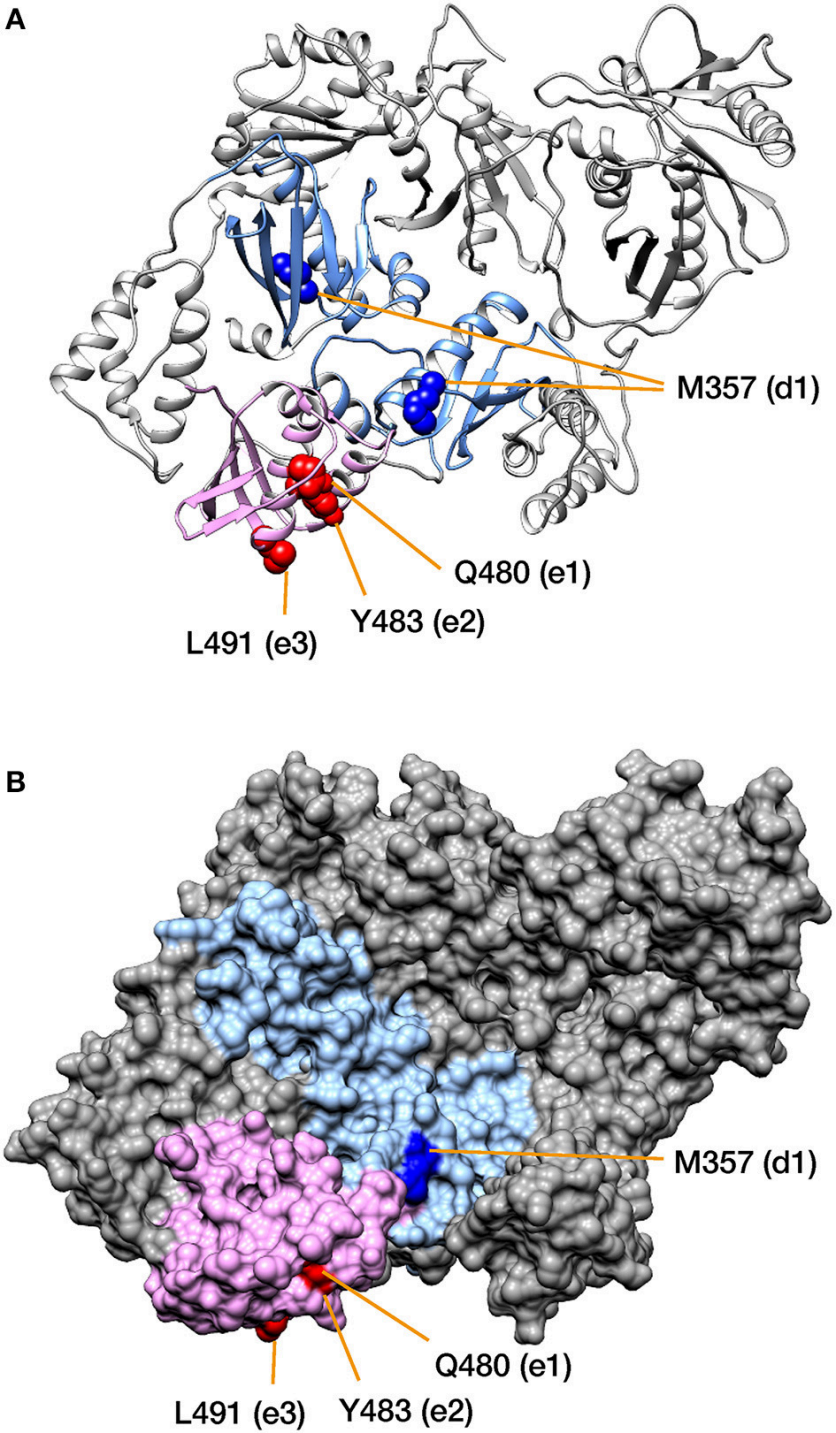
Mapping the amino acid residues corresponding to the differences among HIV-1 subtypes onto RT. The structure of the HIV-1 RT p66/p51 heterodimer is shown in **(A)** as a ribbon diagram, and in **(B)** as a molecular surface representation. The RT connection domain (on p66 and p51) and the RNase H domain (on p66) are colored light blue and light pink, respectively. High-CRE positions d1 and e1-3 are colored blue and magenta, respectively (see also Figure 4). Note that the positions are represented on a space-filling model in panel **(A)**. PDB ID: 1REV.

To localize the regions that characterize the subtypes on the tertiary structure of RT, the aforementioned two domains and the high-CRE sites were mapped onto the three-dimensional conformation of HIV-1 RT (Figure 5). HIV-1 RT is a heterodimer, consisting of p66, which contains the RNase H domain, and p51 without the RNase H domain. The high-CRE sites M357 in the p66 chain of the RT connection domain and Q480, Y483, and L491 in the RNase H domain are physically close (Figure 5A). These amino acid residues were also located on the surface of RT in a representation of the molecular surface structure (Figure 5B and see section Discussion).

## Discussion

In this study, we successfully visualized the similarity relationships of thousands of sequences and summarized these data in SSNs. In the network constructed using the full-length amino acid sequence of the HIV-1 Pol protein, the sequence relationships of the three HIV-1 group M subtypes were visualized, and stepwise clustering divided the subtype A sequence into further clusters (Figure 1). This cluster division corresponded to the different regions in which the viruses were sampled. Therefore, although HIV-1 is prevalent throughout the world, sequence changes arise particularly frequently in epidemic regions, and the network structure properly reflected these differences in their sequences. By comparing the network structures of each domain and the mean CRE values, we identified the RT connection domain and the RNase H domain of RT as characterizing the differences among the HIV-1 subtypes. By calculating the CREs for the amino acid sequences of the connection domain and the RNase H domain, we identified one amino acid residue in the connection domain and three residues in the RNase H domain as subtype-characterizing sites.

Using SSNs, we have previously determined the similarity relationships among a huge number of sequences with complex phylogenetic relationships and have discussed their evolution, including the bacterial CRP/FNR transcriptional regulator superfamily (Matsui et al., 2013) and the novel tRNA genes that have expanded in certain species of eukaryotes (Hamashima et al., 2015). In these reports, we estimated a possible evolutionary pathway by observing the phylogenies inferred from the stepwise analysis of cluster hierarchies. Although, those studies dealt mainly with the amino acid sequences of whole proteins or the nucleotide sequences of whole tRNA genes, in the present study, we constructed SSNs based on domain-level sequences for the first time. In Figure 2Bd, the RT connection domain of subtype A is divided further into two different groups. As described below, at least at the domain level, the difference in the RT activity corresponds to the difference between subtypes B and C. Thus, we speculated that there might be a difference in the RT enzymatic activity between these two groups of the RT connection domain of subtype A. Our results suggest that comparing the networks based on individual protein domains allows not only the detection of subtype differences, but also the functional divergence of the domains analyzed.

We excluded the retroviral aspartyl protease region from the analysis for the reasons described above (see section Domain-Based Network Analysis Shows That RNase H Domain and RT Connection Domain Are Important for Subtype Differentiation). However, comparisons of the network structures (Figure 2) and the mean CRE values (Figure 3) for each domain indicated that the retroviral aspartyl protease region is also a subtype-distinguishing region, in addition to the RT connection domain and RNase H domain. HIV-1 protease has been reported to have different activities and different target cleavage sites, predominantly in subtypes C and B (Velazquez-Campoy et al., 2001; de Oliveira et al., 2003). Together with the thumb domain, the RT connection domain forms a binding cleft and bridges both the N-terminal polymerase activity region and the C-terminal RNase H domain. RNase H is an enzyme that specifically degrades the RNA strand of DNA/RNA complexes during reverse transcription. It has been reported that differences in replication capacity between subtypes B and C are derived from the differences between the RT connection domain and the RNase H domain (Iordanskiy et al., 2010). This supported our current observations at least at the domain level. In addition, mutations in the connection domain and the RNase H domain are known to change the sensitivity of the virus to anti-HIV-1 drugs (RT inhibitors; Julias et al., 2003; Menéndez-Arias et al., 2011), and these are the same domains of the Pol protein that characterize the subtypes identified in this study.

At the amino acid level, the sites responsible for viral subtype differentiation do not perfectly match the drug-resistance mutations (Ehteshami and Götte, 2008; von Wyl et al., 2010; Menéndez-Arias et al., 2011), but are located very close to them in the RT structure (Supplementary Figures 4A,B). In particular, the drug-resistance mutations R356K, R358K, and A360V (Ehteshami and Götte, 2008; von Wyl et al., 2010; Menéndez-Arias et al., 2011) are located on the surface of the RT domain, close to M357, the position with the highest CRE in the connection domain. It is possible that mutations strongly associated with drug resistance are also closely related to the activity of RT, and the domain structure may be greatly altered and its activity reduced by a mutation that changes an amino acid to one with dissimilar biochemical properties. The three resistance mutations are conservative, including from arginine (R) to lysine (K) or from alanine (A) to valine (V). Therefore, we speculate that the HIV-1 subtypes were differentiated by the accumulation of mutations in the surface region where the sequence conservation is low, but at positions located very close to the critical amino acid residues required for enzymatic activity, changing the local structure and modulating the enzyme's activity. In contrast, RT is a heterodimer comprised of subunits p66 and p51. The larger p66 subunit contains the RNase H domain and the catalytic region with the main polymerase activities, whereas the smaller p51 subunit mainly plays a structural role (Telesnitsky and Goff, 1997). In this context, further analysis is required to identify the aspect (activity or structure) of the protein that most strongly affects subtype differentiation. Mutation Q509L, a drug-resistance mutation site in the RNase H domain (Ehteshami and Götte, 2008; Menéndez-Arias et al., 2011), is not physically close to any of the high-CRE sites (Supplementary Figure 4A). Instead, three amino acid residues critical for RNase H activity, D443, E478, and D498, are located together in the RNase H domain close to the high-CRE regions (Supplementary Figure 4C). Mutation E478, in particular, is located very close to Q480, one of three high-CRE sites. We also noted that in the structure of RT complexed with the DNA duplex, M357 is physically close to the DNA molecule but does not interact directly with it (Supplementary Figure S5). Again, these results suggest that the region that characterizes the subtypes is located in the vicinity of the amino acid site responsible for its enzyme activity or RT function.

By mapping the high-CRE sites onto sequence alignments, we found that locations with low sequence conservation do not necessarily characterize the differences in the subtypes (Figure 4). The amino acid residues at the high-CRE sites are conserved within each subtype, but differ between subtypes, so these sites are not fully conserved through all HIV-1 subtypes. This suggests that a region in which CRE is high can accommodate incoming mutations but has functional constraints that do not allow completely random mutations. We found that three HIV-1 subtypes can be distinguished by one amino acid residue (position 357) in the RT connection domain. However, each of the three amino acid residues (positions 480, 483, or 491) in the RNase H domain can be distinguished in only two of the three subtypes, indicating that all three amino acid residues need to be considered to effectively classify the subtypes in this case. We cannot completely exclude the possibility that the accumulation of mutations in these subtype-characterizing regions of the HIV-1 Pol protein is caused by genetic drift. However, it is possible that these mutations are adaptions to the environment at places throughout the world in which HIV-1 is prevalent. The internal environments of various hosts are considered to differ among regions, based on race, the immune system, and the indigenous microbial flora. As seen in Figure 1E, HIV-1 even differs between regions in which the same subtype predominates in the populations. Therefore, we suggest that the virus does not mutate precisely in genomic regions encoding enzyme activities but in neighboring regions. This modulates the enzyme functions to allow the adaptation of the virus to geographic regional differences it encounters in areas of prevalence.

Many currently emerging viruses that cause pandemics throughout the world are RNA viruses characterized by high mutation rates, including *Ebola virus, Zika virus*, and others. Genome analyses have already shown that as viral infections spread, mutations accumulate in the viral genomic sequences, causing them to differ in different endemic areas (Simon-Loriere et al., 2015; Tong et al., 2015; Metsky et al., 2017). Our research provides a molecular basis for HIV-1 evolution and subtype differentiation, and should extend our understanding of the evolution and differentiation of other RNA viruses, including emerging viruses.

## Author contributions

SN and AK conceived and designed the study, and SN wrote the manuscript. SN, JI, and GM performed the analyses and interpreted the data. AK and MT edited the manuscript. AK supervised the project. All the authors have read and approved the final manuscript.

### Conflict of interest statement

The authors declare that the research was conducted in the absence of any commercial or financial relationships that could be construed as a potential conflict of interest.

## Abbreviations

HIV-1: *Human immunodeficiency virus 1*
RT: reverse transcriptase
SSN: sequence similarity network
CRE: cumulative relative entropy.
