# Supplementary material for: Evolutionary Analysis of HIV-1 Pol Proteins Reveals Representative Residues for Viral Subtype Differentiation
Source: Front Microbiol. 2017 Nov 2;8:2151. doi: 10.3389/fmicb.2017.02151 (PMC5666293; doi:10.3389/fmicb.2017.02151)
Supplement: Supplementary file 1 [file Image1.PDF]

Supplementary Material

Evolutionary analysis of HIV-1 Pol proteins reveals representative residues for viral subtype differentiation

Shohei Nagata[1,2], Junnosuke Imai[1,3], Gakuto Makino[1], Masaru Tomita[1,2,3], Akio Kanai[1,2,3*]

[1]Institute for Advanced Biosciences, Keio University, Tsuruoka, Japan
[2]Faculty of Environment and Information Studies, Keio University, Fujisawa, Japan
[3]Systems Biology Program, Graduate School of Media and Governance, Keio University, Fujisawa, Japan

*** Correspondence:** Akio Kanai: akio@sfc.keio.ac.jp

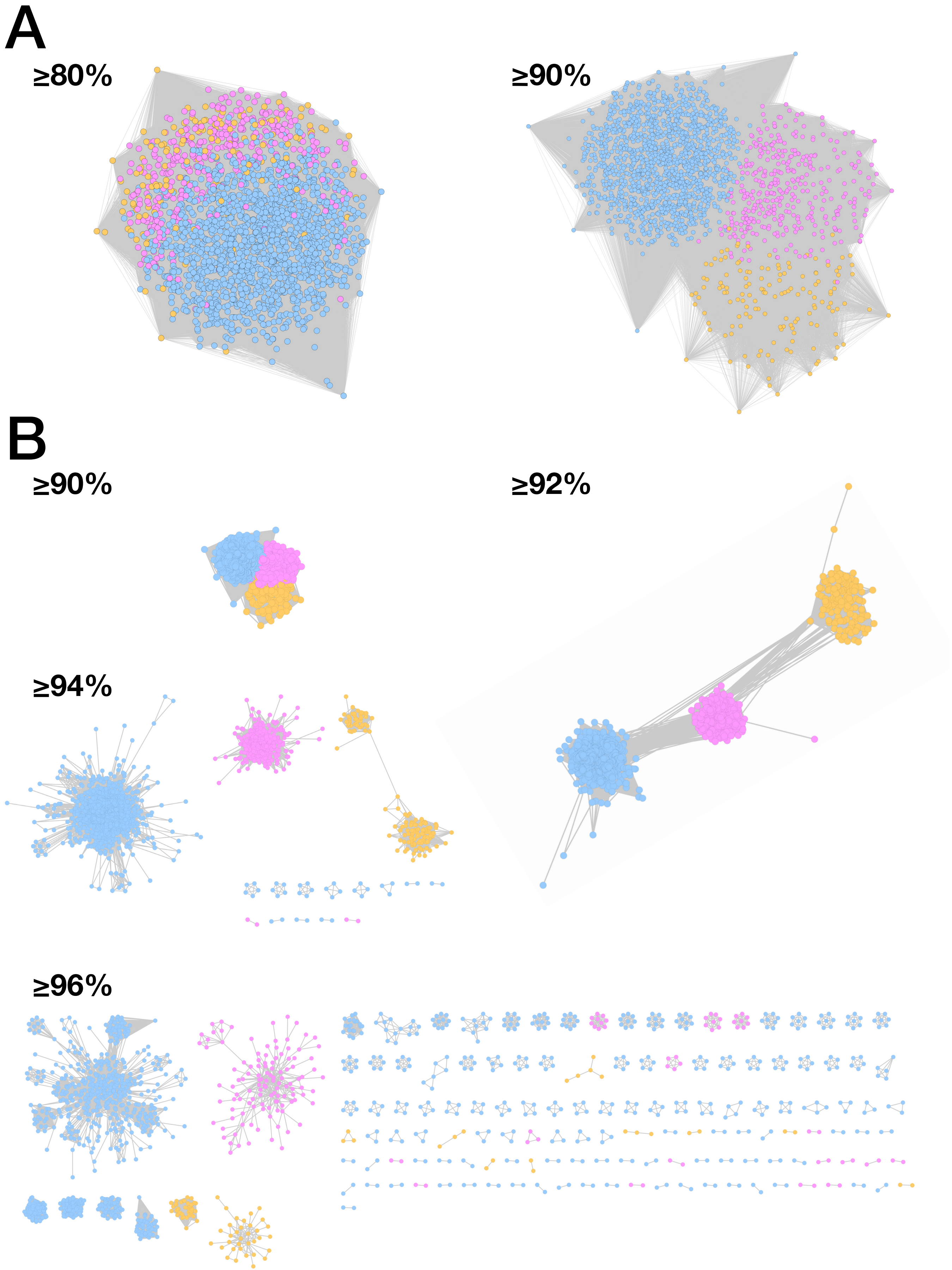

Supplementary Figure S1. Changes in the network structure when a series of sequence similarity thresholds is applied. A total of 2,052 amino acid sequences of the HIV-1 Pol protein were classified based on their sequence similarities. Nodes (colored dots) represent each Pol protein sequence and the edge lengths represent the sequence similarities. Nodes are linked by an edge when the sequence identity is above the threshold. The drawn node size in panel B is enlarged six times relative to that in panel A.

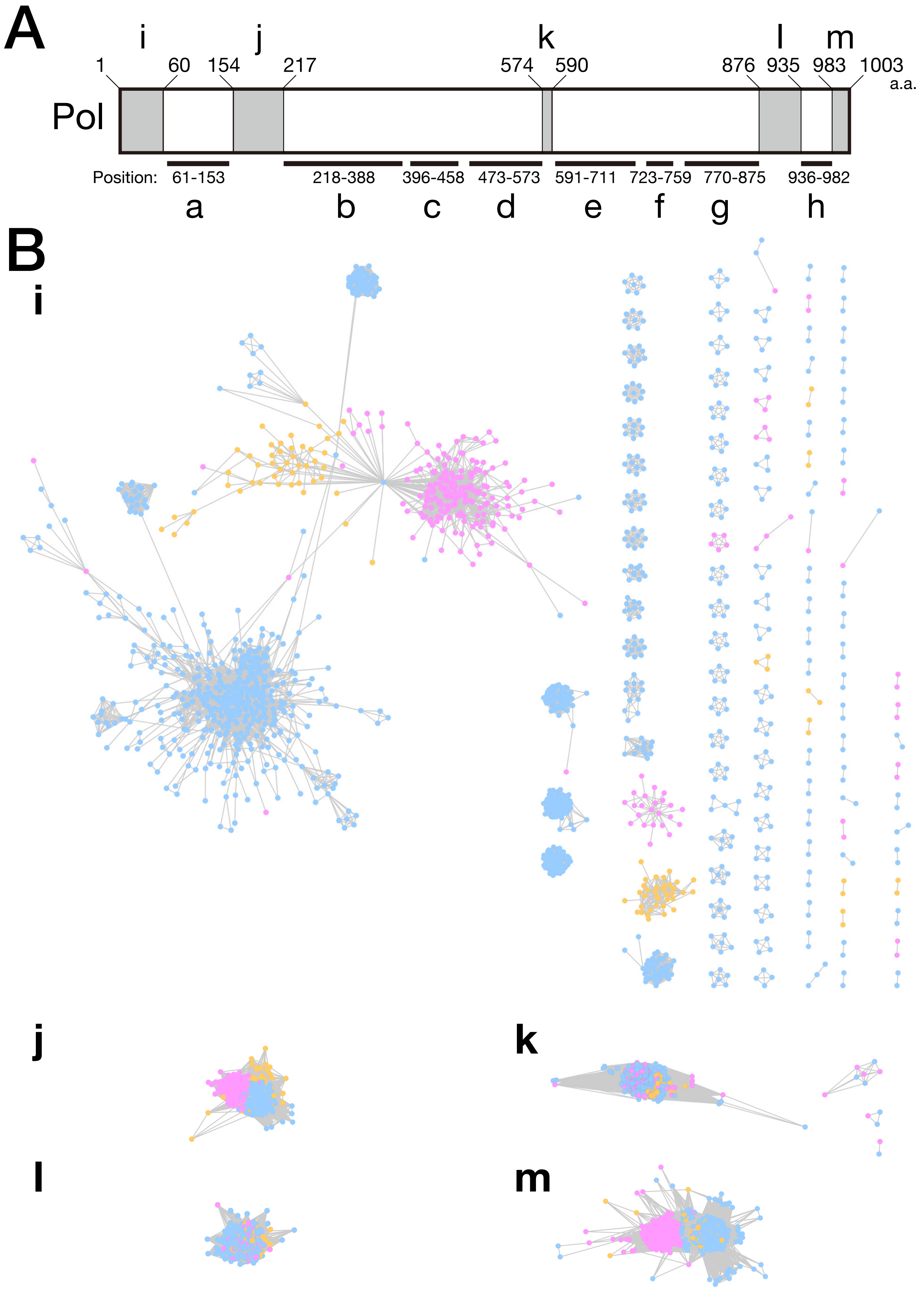

Supplementary Figure S2. Comparisons of the SSNs of non-domain regions in the HIV-1 Pol protein. (A) Schematic representation of the HIV-1 Pol protein showing the positions and lengths of the functional domains (a–h) and the regions outside the domains (i–m). **(B)** SSNs of each non-domain region (i–m) in the HIV-1 Pol protein (2,052 sequences) were created and colored according to subtype. Nodes (colored dots) represent the functional domain sequences and the edge lengths represent the sequence similarities. Symbols (i–m) correspond to those in panel A.

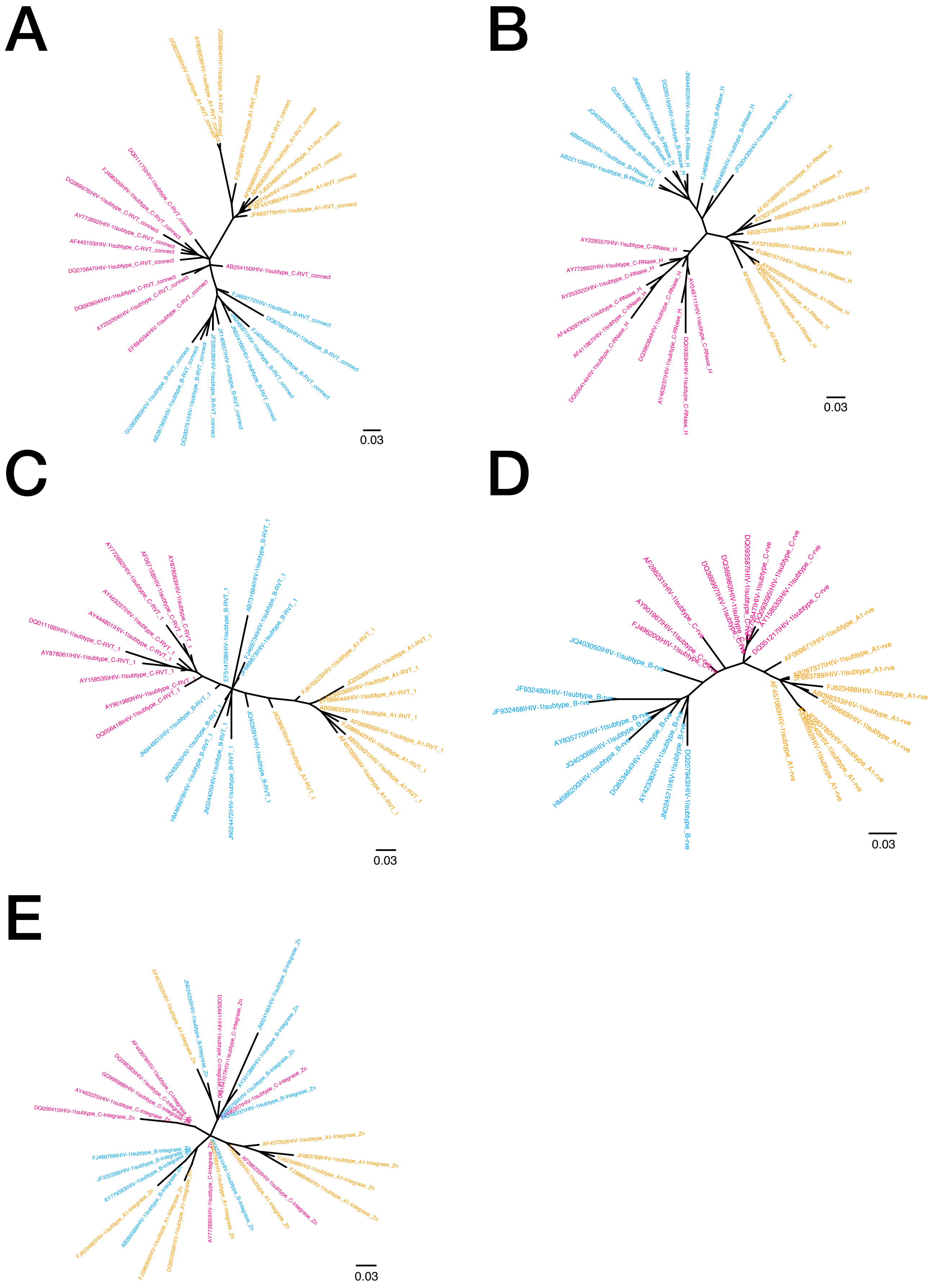

Supplementary Figure S3. Phylogenetic trees of functional domains of the HIV-1 Pol protein. Maximum likelihood phylogenetic trees of each functional domain of the HIV-1 Pol protein: A, RT connection domain; B, RNase H; C, RT (RNA-dependent DNA polymerase); D, integrase core domain; E, integrase zinc-binding domain. Ten domain sequences were randomly selected from each subtype (subtypes A, B, and C) and used to calculate the trees. Scale bar indicates the number of substitutions per site.

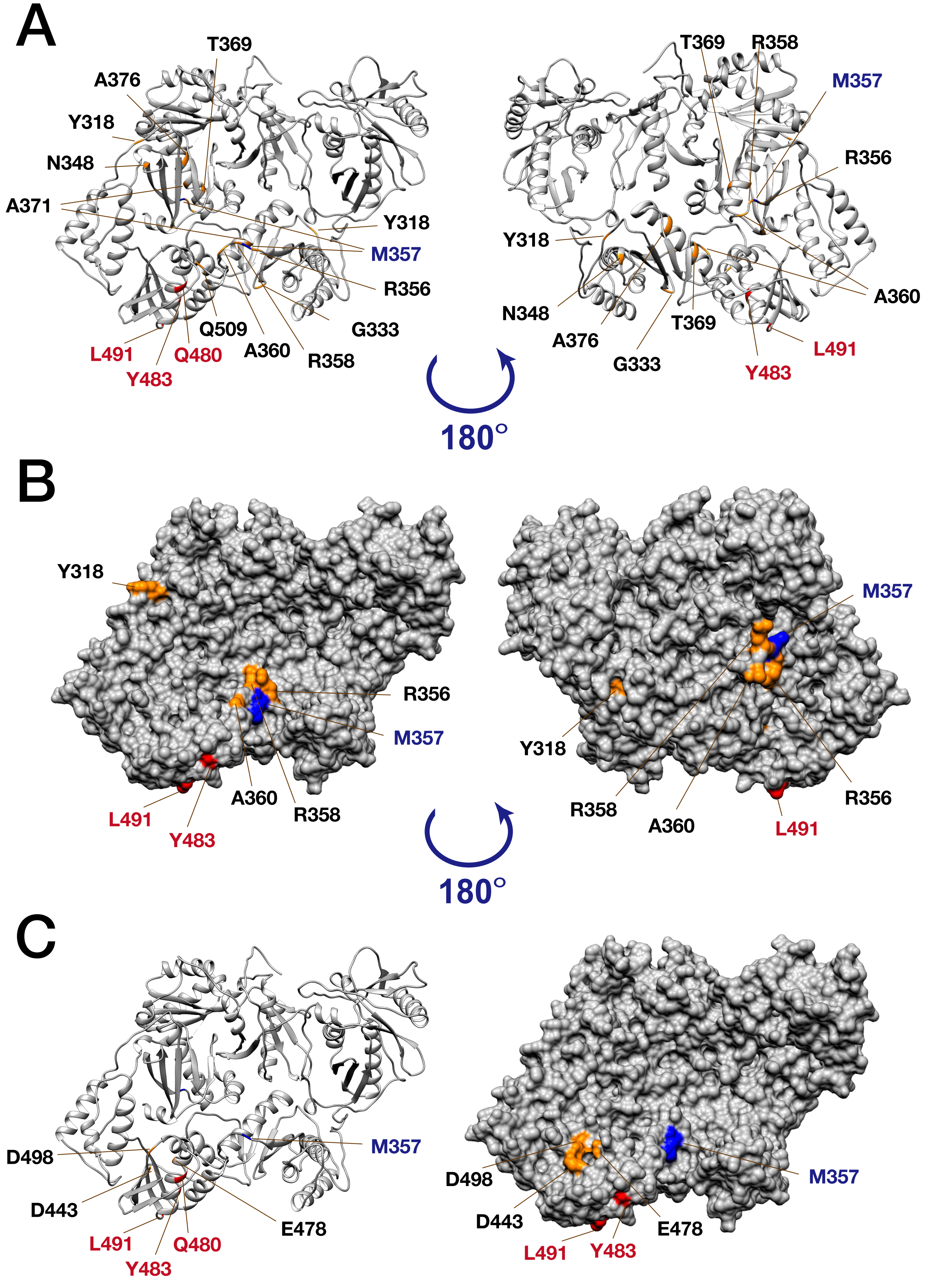

Supplementary Figure S4. Mapping drug-resistance-associated mutations and enzyme active sites in RT. (A, B) Drug-resistance-associated mutations are shown in the structure of the HIV-1 RT p66/p51 heterodimer, represented as a ribbon diagram **(A)**, and a molecular surface representation **(B)**. Mutations associated with resistance to RT inhibitors (according to following references: Ehteshami and Götte, 2008; Menéndez-Arias et al., 2011; von Wyl et al., 2010) are colored orange. **(C)** Enzyme active sites of the RNase H domains are mapped and colored orange. See also Figure 5 for details. PDB ID: 1REV.

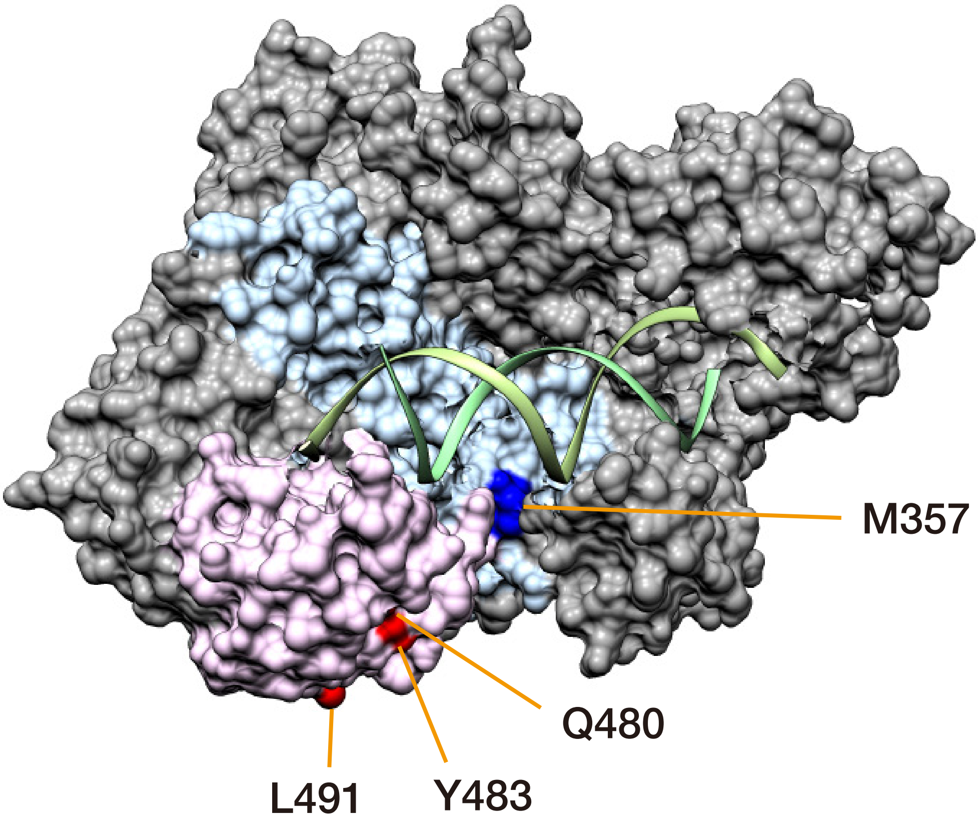

Supplementary Figure S5. Mapping the amino acid residues corresponding to the differences among HIV-1 subtypes onto RT complexed with the DNA duplex. The structure of the HIV-1 RT p66/p51 heterodimer complexed with the DNA duplex is shown as a molecular surface representation. The DNA molecules are represented as green-colored ribbon diagrams. See also Figure 5 for details. PDB ID: 3KJV.
